# Supplementary material for: Altered microRNA Transcriptome in Cultured Human Liver Cells upon Infection with Ebola Virus
Source: Int J Mol Sci. 2021 Apr 6;22(7):3792. doi: 10.3390/ijms22073792 (PMC8038836; doi:10.3390/ijms22073792)
Supplement: Supplementary file 1 [file ijms-22-03792-s001.zip › Supplementary_File/D_ KEGG_Analysis_Results/16-30nt_pathway_Makona-24h-Huh7_vs_Control-24h-Huh7_down.mature_mirna_targets/hsa_EnrichmentScore.pdf]

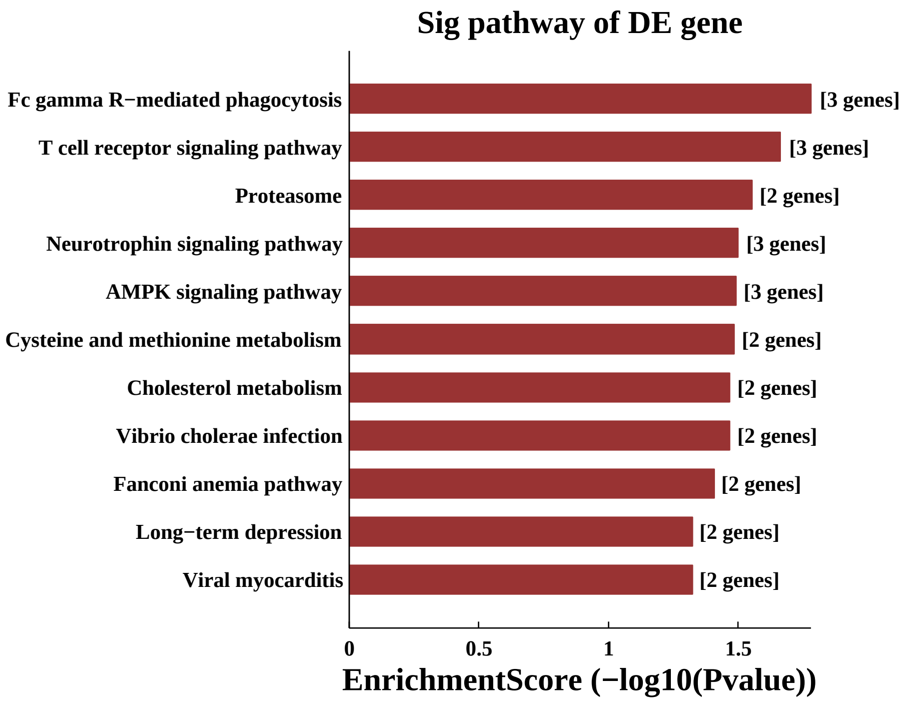
Sig pathway of DE gene
Fc gamma R−mediated phagocytosis
T cell receptor signaling pathway
Proteasome
Neurotrophin signaling pathway
AMPK signaling pathway
Cysteine and methionine metabolism
Cholesterol metabolism
Vibrio cholerae infection
Fanconi anemia pathway
Long−term depression
Viral myocarditis
[3 genes]
[3 genes]
[2 genes]
[3 genes]
[3 genes]
[2 genes]
[2 genes]
[2 genes]
[2 genes]
[2 genes]
[2 genes]
0
0.5
1
1.5
EnrichmentScore (−log10(Pvalue))
